# Mycorrhizosphere Bacterial Communities and their Sensitivity to Antibacterial Activity of Ectomycorrhizal Fungi

**DOI:** 10.1264/jsme2.ME18146

**Published:** 2019-05-11

**Authors:** Makoto Shirakawa, Iwao Uehara, Megumi Tanaka

**Affiliations:** 1 Graduate School of Agriculture, Tokyo University of Agriculture 1–1–1 Sakuragaoka, Setagaya, Tokyo, 156–8502 Japan; 2 Faculty of Regional Environment Sci., Tokyo University of Agriculture 1–1–1 Sakuragaoka, Setagaya, Tokyo, 156–8502 Japan

**Keywords:** antibacterial assay, *Paraburkholderia*, *Bacillus*, *Suillus bovinus*, *Pinus densiflora*

## Abstract

We investigated whether ectomycorrhizal (ECM) fungal species exhibit antibacterial activity towards culturable bacterial communities in mycorrhizospheres. Four hundred and thirty bacterial strains were isolated from the ECM root tips of *Pinus densiflora* and bulk soil, and 21 were co-cultured with six ECM fungal species. Three hundred and twenty-nine bacterial 16S rDNA sequences were identified in ECM roots (*n*=185) and bulk soil (*n*=144). Mycorrhizosphere isolates were dominated by Gram-negative *Proteobacteria* from 16 genera, including *Burkholderia*, *Collimonas*, *Paraburkholderia*, and *Rhizobium*. *Paraburkholderia* accounted for approximately 60%. In contrast, bulk soil isolates contained a high number of Gram-positive *Firmicutes*, particularly from *Bacillus*. *Paraburkholderia* accounted for ≤20% of the bacterial isolates from bulk soil, which was significantly lower than its percentage in ECM root tips. Co-cultures of six ECM fungal species with the 21 bacterial strains revealed that eight strains of three Gram-positive genera—*Arthrobacter*, *Bacillus*, and *Lysinibacillus*—were sensitive to the antibacterial activity of the fungi. In contrast, the Gram-negative strains, including five *Paraburkholderia* strains, two *Burkholderia* strains, and a *Rhizobium* sp., were not sensitive. The strength of fungal antibacterial activity varied in a species-dependent manner, but consistently affected Gram-positive bacteria. These results suggest that Gram-positive bacteria are excluded from the mycorrhizosphere by the antibacterial activity of ECM fungi, which develops specific soil bacterial communities in the mycorrhizosphere.

Symbiotic microorganisms are important factors in plant growth and health, and, thus, a plant may be regarded as a holobiont with its microflora (45). Ectomycorrhizal (ECM) fungi exist in a symbiotic relationship with tree roots and are ubiquitous in temperate forest ecosystems (37). These fungi contribute to the growth of the host tree by facilitating the uptake of water and nutrients and improving the plant’s resistance to biotic and abiotic stress (11, 37, 43). They also promote distinctive morphological changes, such as the development of mantle and external hyphae around the tree root, and form a specific environment termed the “ectomycorrhizosphere” (24). The mycorrhizosphere consists of ECM fungal mycelia on the surface of the root and the surrounding soil area. Inter- and intracellular areas of the mantle and Hartig net are colonized by various bacterial species (30). These bacterial communities differ from those in the surrounding soil (42) and exhibit a greater ability to solubilize inorganic nutrients than non-mycorrhizosphere bacteria (7, 40). Additionally, certain strains inhabiting the mycorrhizosphere, including those of *Burkholderia*, *Pseudomonas*, and *Rhizobium*, have been reported as mycorrhiza helper bacteria (MHB), which promote the formation of mycorrhizae (9, 17). Therefore, the mycorrhizosphere provides a niche for specific bacteria, which, in turn, play an important role in the symbiotic system of trees.

Studies using molecular and culturing methods revealed that ECM fungi affect the composition of bacterial communities in the mycorrhizosphere (6, 19, 28). Marupakula *et al*. (25) reported that the compositions of bacterial communities depend on specific fungal species. However, Uroz *et al*. (41) demonstrated the presence of similar bacterial communities in the mycorrhizospheres of two different ECM fungal species. In both of these studies, bacterial genera including *Burkholderia*, *Bradyrhizobium*, or both were detected at high levels; similar findings were reported in other ECM studies (5, 28). Therefore. ECM fungi do not appear to be the main factor influencing the mycorrhizosphere bacterial community, but may contribute to the creation of a preferred environment for specific bacteria.

The focus of the present study was to investigate the effects of the antibacterial activity of ECM fungi on culturable bacterial community formation in the mycorrhizosphere. ECM fungi produce various antimicrobial substances (13, 21, 39), and their effects on bacteria have been reported through *in vitro* studies using extracts from the sporocarp (2, 29) or mycorrhizae (22) as well as pure cultures of mycelia (44). However, these studies used model microorganisms, such as *Bacillus subtilis*, *Escherichia coli*, *Staphylococcus aureus*, and *Pseudomonas aeruginosa*; few studies have focused on bacterial strains that inhabit the mycorrhizosphere (2, 3, 15). This antibacterial activity has also been observed in the mycorrhizae *in situ*; Olsson *et al*. (33) showed that ECM hyphae in soil reduce the activity of soil bacteria. Thus, the antibacterial activity of ECM fungi may exert selective pressure on bacteria in the mycorrhizosphere (6); however, their influence on the formation of culturable bacterial communities in the mycorrhizosphere remains poorly understood.

To clarify whether the antibacterial activity of ECM fungi influences culturable mycorrhizosphere bacterial community formation, two hypotheses were examined. The first is that ECM fungi have a common influence on the culturable mycorrhizosphere bacterial community regardless of species differences. The second is that the culturable bacterial community in the mycorrhizosphere mainly consists of bacteria that are not sensitive to the antibacterial activity of ECM fungi. To test these hypotheses, we isolated and analyzed bacterial strains from the mycorrhizosphere of *Pinus densiflora* and from bulk soil. The sensitivities of these isolated bacterial strains to the activities of six ECM fungal species were evaluated using a co-culture analysis.

## Materials and Methods

### Site and sampling

ECM root tips and bulk soil samples were collected in a temperate secondary forest in Ome city, Tokyo, Japan (35°47′50.4″N 139°15′44.4″E). The mean annual temperature recorded at the site was 14.4°C, and annual precipitation was 1412.5 mm (Japan Meteorological Agency. 2017. https://www.data.jma.go.jp/obd/stats/etrn/view/annually_a.php?prec_no=44&block_no=1001&year=2017&month=&day=&view=p). The site was dominated by *Quercus serrata* and *P. densiflora* was locally abundant. In addition to these plants, several ECM tree species, including *Q. myrsinifolia* and *Castanea crenata*, have been confirmed. We selected *P. densiflora*, and four sites containing *P. densiflora* were established and labeled A, B, C, and D. Sampling sites A and B consisted of mixed stands of *Q. serrata* and *P. densiflora*, with both sites facing a path. Site C featured a gap due to periodic weeding for the management of planted pine shrubs. Site D was composed of *P. densiflora* and *Chamaecyparis obtusa* located along a ridge. Sampling sites were separated from each other by at least 200 m (gradient distance) (Table 1).

Sampling of the pine root system and bulk soil was performed in May, June, and August 2016 and in October 2017. Two to five mature pine trees were selected at each site, and five naturally grown pine seedlings were selected from sampling site C only (Table 1). Trees were chosen that were at least 4 m from each other. To avoid contamination from other tree roots, we traced lateral roots from each selected tree trunk to obtain a 15–30-cm-long root system sample from within a radius of 1 m and depth of 5–15 cm. Bulk soil was sampled 1 m from each tree trunk as a soil core (5×5×10 cm, excluding litter). We sampled 18 pine root systems from 13 trees, five pine seedlings, and 18 bulk soil cores. These samples were stored at 4°C and processed within one week. A bulk soil core was also collected from five random locations in each sampling site for physical and chemical analyses.

### Soil chemical and physical properties

Soil hardness (SH) and litter thickness (LH) were measured using the Yamanaka-type soil hardness tester (Fujiwara Scientific, Tokyo, Japan) and ruler, respectively, during soil core collection. Soil water content (SWC) was measured on the basis of weight, with 30 g dried at 60°C for 48 h and reweighed. Soil pH was measured with a model IM 32-P pH meter (DKK-Toa, Tokyo, Japan) following the addition of 25 mL of distilled water to 5 g of dried sample soil (<0.5 mm). After shaking for 30 min, the suspension was left for 30 min prior to the recording of pH with a pH electrode. The ratio of carbon to nitrogen (C/N) was measured by the dry combustion method using an MT-700 Mark II C/N analyzer (Yanaco Technical Science, Tokyo, Japan). Soil properties at the four sites are shown in Table 1.

### Bacterial isolation

The sampled root systems were washed gently in tap water with a brush under a stereomicroscope. ECM root tips were carefully removed from the root system and sorted into morphotypes based on size, shape, surface color, texture, and emanating hyphae (1, 18). Eight adjacent root tips per mycorrhizal morphotype were then isolated from each root system. Each sample was transferred to 1 ml of sterile water and shaken for one min using a VORTEX Genius 3 (IKA Japan K.K., Osaka, Japan). This process was repeated five times to remove soil particles and debris. Three of the eight root tips were stored at –30°C for the molecular identification of ECM fungi, and the remaining five were homogenized using a micropestle and suspended in 1 mL of sterile water. The stock solutions obtained were serially diluted (10^–1^ to 10^–4^), and 100 μL of each dilution was spread onto yeast glucose (YG) agar medium (38). All plates were incubated at 24–25°C for three to seven d in the dark, and those contaminated with saprotrophic fungi were discarded. Ten bacterial colonies per mycorrhizal morphotype were randomly selected for analysis. Bacteria were also isolated from bulk soil. Briefly, fine roots and litter were removed from each soil sample, and 0.15 g of the sample was suspended in 1.35 mL of sterile water. One hundred microliters from each serial dilution (10^–1^ to 10^–6^) of the stock solution was spread on YG agar medium. Culture plates were incubated and bacterial isolates for analysis were selected as described above. The community compositions of the selected bacterial isolates were analyzed, and 21 strains were subjected to bioassay experiments after 16S ribosomal RNA gene (rDNA) sequencing.

### DNA extraction, amplification, and sequencing

The V1–V6 region of bacterial 16S rDNA was amplified by direct PCR from a single bacterial colony using EmeraldAmp^®^ PCR Master Mix (Takara Bio, Shiga, Japan) with the forward primer 8F 5′-AGAGTTTGATCCTGGCTCAG-3′ and reverse primer 1400R 5′-CGGTGTGTACAAGGCCC-3′. PCR amplification was performed using a Thermal Cycler Dice^®^ Gradient (Takara Bio) under the following conditions: 94°C for 1 min; 40 cycles of 98°C for 10 s, 55°C for 30 s, and 72°C for 90 s; and 72°C for 7 min. PCR products were then confirmed using 1.2% agarose gel electrophoresis. The amplified products were purified using ExoSAP-IT^TM^ (Thermo Fisher Scientific, Tokyo, Japan) and directly sequenced with the BigDye^®^ Terminator v3.1 (Thermo Fisher Scientific using the primer 1100R 5′-GGGTTGCGCTCGTTG-3′. Sequences longer than 300 bp were identified with specific bacterial genera based on >97% homology in BLAST results against the DNA Database of Japan (DDBJ) or the National Center for Biotechnology Information (NCBI) database.

ECM fungal DNA was extracted from dried ECM root tips using the cetyltrimethyl ammonium bromide method (27). PCR was performed to amplify the internal transcribed spacer (ITS) region of rDNA using EmeraldAmp^®^ PCR Master Mix and the primers ITS1F and ITS4. PCR amplification was performed as described above. The amplified products were purified using ExoSAP-IT^TM^ and subjected to direct sequencing using BigDye^®^ Terminator v3.1 and ITS1 as the sequencing primer. Fungal ITS sequences were assembled into molecular operational taxonomic units (MOTUs) with >97% similarity using ATGC (ver. 7.0; GENETYX Corp., Tokyo, Japan). Each MOTU (>300 bp) was subjected to a BLAST search against the DDBJ or NCBI database and grouped accordingly (≥97% similarity for species level, ≥95% for genus level, ≥90% for family level, and <90% for order or higher taxonomic levels). The reading of the base sequences of positive amplicons was entrusted to the Center for Omics and Bioinformatics, the University of Tokyo. The 16S rDNA and ITS sequences identified were deposited in the DDBJ database under the accession numbers LC435746–LC436075 and LC436076–LC436097, respectively.

### Bacterial and ECM fungal strains used for bioassay experiments

**Fig. 1:**
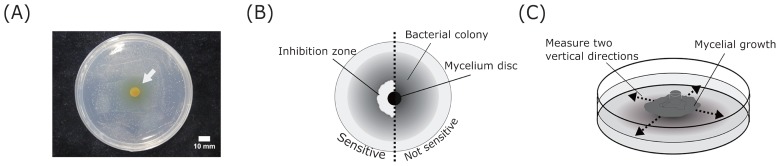
Antibacterial assay; co-cultivation of ECM fungi and bacteria. (A) *Suillus bovinus* exhibited antibacterial activity against *Bacillus drentensis* strain S-s330 in a preliminary test. The white arrow indicates the formation of an inhibition zone around a mycelial disc. (B) The presence or absence of inhibition zones. (C) Measurement of mycelial growth after cultivation for four weeks.

Twenty-one isolated bacterial strains—eight from the ectomycorrhizae of *P. densiflora* and 13 from bulk soil—were used for bioassay experiments. All strains were identified to the genus level based on 16S rDNA sequencing (average sequence length of 691 bp, including the V3–V5 regions). Eleven strains were identified as Gram-positive (*Arthrobacter*, *Bacillus*, *Lysinibacillus*, or *Paenibacillus*) and the remainder as Gram-negative (*Burkholderia*, *Collimonas*, *Massilia*, *Paraburkholderia*, or *Rhizobium*). These bacterial genera have been isolated in previous studies involving the rhizosphere, its associated environment, or both (10, 28, 31, 40); some of these strains have also been reported as MHBs (9, 16). All bacterial strains were grown at 25°C on YG agar medium for three d in the dark and then subjected to sensitivity testing. *B. drentensis* strain S-s330 was used as a preliminary test strain to select ECM fungi for co-cultivation testing. This strain—isolated from rhizosphere soil—exhibited sensitivity to the antimicrobial activity of *Suillus grevillei* (36) (Fig. 1A) and was provided by the Laboratory of Silviculture, Tokyo University of Agriculture.

Thirty-three ECM fungal strains were provided by Prof. K. Nara, the University of Tokyo. Six were selected for antibacterial assays based on the relative sizes of inhibition zones achieved through co-cultivation testing with *B. drentensis* strain S-s330. Specifically, large zones of inhibition were observed with *S. bovinus* and *Hebeloma mesophaeum*, small zones of inhibition with *Rhizopogon roseolus* and *Russula mariae*, and no inhibition with *Amanita pantherina* and *S. granulatus* (Table S1). These fungal strains were pre-cultured on half-strength Modified Melin-Norkrans (1/2 MMN) agar medium (26) in the dark for approximately one month at 20°C.

### Antibacterial assay

The antibacterial activities of the six ECM species were assessed by co-culture testing with 21 bacterial strains. The antibacterial assay was performed on 90-mm petri dishes containing 20 mL of 1/2 MMN agar medium. Suspensions of the bacterial strains were adjusted to an optical density at 600 nm (OD_600_) of 0.05 with sterile water, and 50 μL of each suspension was spread on 1/2 MMN agar medium. Six-millimeter discs containing ECM fungal mycelia were placed onto the center of the agar. After an incubation in the dark at 20°C for three d, the bacterial colonies that formed on the medium were observed and the diameter of the inhibition zone was measured (Fig. 1B). After a further incubation for four weeks, the growth of mycelia was measured in two perpendicular directions and mean radial growth obtained (Fig. 1C). Three replicates of each co-culture system were performed.

### Statistical analysis

Statistical analyses were performed using R version 3.2.2 (R Core Team, 2015; https://www.r-project.org/) and IBM SPSS statistics21 (IBM, Armonk, NY, USA). Soil environmental factors, including SH, LH, SWC, pH, and the C/N ratio, in the four sampling sites were compared using a one-way analysis of variance (one-way ANOVA) and Tukey’s test. The relative proportions of culturable bacteria from ECM root tips and bulk soil samples were compared using Fisher’s exact test. Non-metric multidimensional scaling ordination (NMDS) was performed based on the number of occurrences at the genus level per sample using the Bray-Curtis dissimilarity measure to visualize the composition of the culturable bacterial community. Soil environmental factors in the four sampling sites were also fitted in ordination using the “envfit” function of the vegan package. Comparisons between the culturable bacterial communities of ECM root tips and bulk soil were performed using a permutational multi-variate analysis of variance (PERMANOVA). The Student’s *t*-test was used to analyze the mycelial growth of each fungal strain co-cultured with the 21 strains of bacteria relative to the control.

## Results

### Culturable bacterial communities

In the present study, 18 pine root samples—13 from trees and five from seedlings—and 18 bulk soil samples were collected from sites A, B, C, and D. Among these samples, 430 bacterial colonies were isolated and 25 ECM morphotypes were identified. Overall, the sequence analysis revealed 329 bacterial 16S rDNA sequences (average 690 bp, including the V3–V5 regions) and 22 ECM fungal ITS sequences (average 481 bp). Sequences were excluded from the analysis when less than seven were obtained per ECM morphotype and/or when the ECM fungal species was unknown.

**Fig. 2:**
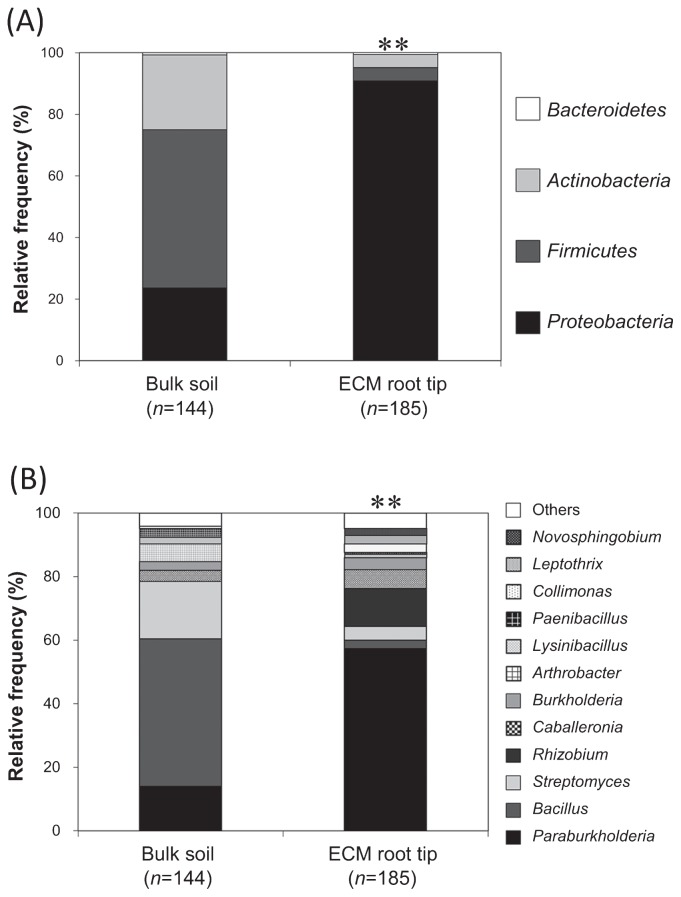
The relative proportions of culturable bacteria from ECM root tips and bulk soil samples (A) at the phylum level and (B) at the genus level. Genera with a frequency of occurrence of ≤3% were classified as “Others”. Fisher’s exact test **, *P*<0.01.

The relative abundance of bacterial genera in ECM root tips was significantly different from that in bulk soil samples (Fig. 2A, B). The 185 16S rDNA sequences (among the total of 329) from ECM root tip-derived bacterial isolates were identified and matched with 16 genera; most (90.8%) were Gram-negative *Proteobacteria*, including *Burkholderia*, *Caballeronia*, *Collimonas*, *Novosphingobium*, *Rhizobium*, and *Paraburkholderia*. One hundred and six (57.3%) of the total of 185 sequences analyzed were accounted for solely by *Paraburkholderia*, a member of the *Betaproteobacteria* group. *Rhizobium*, which belongs to the *Alphaproteobacteria* group, was the predominant genus in only two of the 22 ECM root tips. The remaining 144 rDNA sequences of bulk soil-derived bacterial isolates were identified and matched with 14 genera, including *Bacillus*, *Streptomyces*, *Arthrobacter*, and *Paenibacillus*. In contrast to those associated with ECM root tips, 75.7% of these isolates were Gram-positive. The predominant microbes isolated from bulk soil were *Bacillus* (46.5%) followed by *Streptomyces*. *Paraburkholderia* accounted for only 13.9% of bulk soil-derived bacterial isolates, which was markedly lower than the levels found in ECM root tips.

**Fig. 3:**
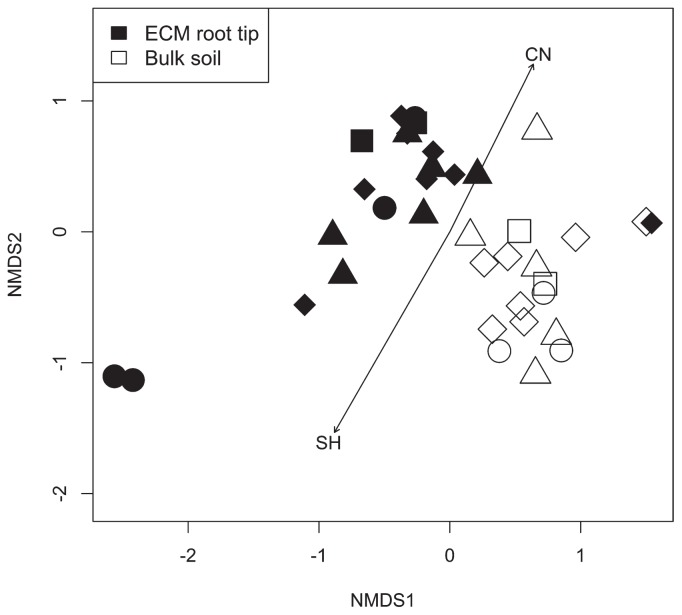
Non-metric multidimensional scaling (NMDS) ordination of the culturable bacterial community composition associated with ECM root tips of *P. densiflora* and bulk soil. Black and white colors represent ECM root tips and bulk soil. Circles, triangles, diamonds, and squares indicate four sampling sites A–D, respectively. The values of the effects of soil environmental factors on bacterial communities, based on environmental fitting tests: SH (R^2^=0.2321, *P*=0.011), LT (R^2^=0.0017, *P*=0.977), SWC (R^2^=0.0346, *P*=0.546), pH (R^2^=0.0029, *P*=0.965), C/N (R^2^=0.1521, *P*=0.050). Stress=0.0969.

NMDS ordination indicated that the culturable bacterial communities of ECM root tips and bulk soil differed, which was confirmed by the PERMANOVA analysis (*P*<0.001; Fig. 3). In contrast, no significant differences were observed between the culturable bacterial communities of the ECM root tips. The 22 ECM root tips represented by A1 to D3 were classified into 18 MOTUs including *Russula* sp., *Sebacina incrustans*, *Tomentella cinerascens*, *Lactarius* sp., and *Rhizopogon* sp. (Table 2). Of these, A1 and A3b; C2a, C2b, and Cs1b; and Cs4a and Cs4b were regarded as common MOTUs *Russula* sp. 1, *Tomentella* sp., and *Rhizopogon* sp. 1, respectively (even though C2a and C2b, and Cs4a and Cs4b were isolated from the same root system, they were analyzed individually due to their different morphological types and bacterial communities). Although the ECM roots possessed different culturable bacterial communities, 19 out of the 22 ECM root tips harbored *Paraburkholderia*. Therefore, we assembled isolates of this genus into a MOTU with >99% similarity using ATGC and re-ordination with NMDS. Nevertheless, *Paraburkholderia* was divided into 13 MOTUs; NMDS ordination did not show a significant difference (*P*>0.05; Fig. S1). Moreover, the soil environmental factors of the sampling sites (Table 1) did not influence culturable bacterial communities in ECM root tips and bulk soil, although a significant influence of SH and C/N was confirmed by the envfit test (SH: R^2^=0.2321, *P*=0.011, C/N: R^2^=0.1521, *P*=0.050) (Fig. 3).

### Antibacterial assay

Table 3 shows the mean diameter of inhibition zones resulting from the antibacterial activities of six ECM fungal strains on 21 bacterial strains. In eight of the strains, a clear halo was observed around the mycelial disc after a 3-d incubation. The strains exhibiting sensitivity were Gram-positive bacteria belonging to the genera *Arthrobacter*, *Bacillus*, and *Lysinibacillus*. Ten Gram-negative strains—five *Paraburkholderia*, two *Burkholderia*, and one each of *Collimonas* sp., *Massilia* sp., and *Rhizobium* sp.—were not sensitive to fungal antibacterial activity. In these strains, the abundant production of extracellular polysaccharide was observed. Moreover, the results of the sensitivity test were consistent regardless of the source (ECM roots or bulk soil) of the isolated bacteria. Overall, the ECM fungal test strains, with the exception of *S. granulatus* and *R. mariae*, displayed variable levels of antibacterial activity. However, they all exhibited inhibitory activity against Gram-positive bacteria only (Table 3). Among ECM strains, *S. bovinus* exhibited the strongest antibacterial activity, with the inhibition of *Bacillus* spp. 1, 2, 4–6, 8, *Arthrobacter* sp., and *Lysinibacillus* sp.; *Bacillus* sp. 4 was the most sensitive. *H. mesophaeum* and *R. roseolus* also exhibited antibacterial activity; the former inhibited six *Bacillus* strains (spp. 1, 2, 4–6, and 8) and the latter, four *Bacillus* strains (spp. 2, 4–6) and *Lysinibacillus* sp. *A. pantherina* displayed the weakest antibacterial activity among all ECM strains tested; only a single bacterial strain of *Lysinibacillus* sp. was found to be sensitive.

The results obtained for the mycelial growth of ECM fungal strains co-cultured with bacteria for four weeks are summarized in Table 4. The inhibition of mycelial growth was significantly greater by bacteria than that on control plates. Mycelial growth was markedly inhibited in co-culture experiments with Gram-negative bacteria with the exception of the co-culture of *S. granulatus* with *Collimonas* sp. Complete mycelial suppression was confirmed in four of the six strains tested. Strong mycelial suppression was observed in co-cultures of *R. mariae* with nine of the ten bacterial strains. In contrast, the mycelial growth of fungal strains co-cultivated with Gram-positive bacteria showed a low level of inhibition; significant levels of inhibition were observed only sporadically relative to that with Gram-negative bacteria. These low levels of inhibition were noted in the treatment of fungal strains that exhibited antibacterial activity, or co-cultured with *Bacillus* spp. 1, 2, 4–6, and 8, and *Lysinibacillus* sp. (excluding *Arthrobacter* sp.). However, similar results were observed with *S. granulatus* and *R. mariae*, which did not exhibit any antibacterial activity (Table 4).

## Discussion

In the present study, culturable bacterial communities in the ECM root tips of *P. densiflora* were significantly different from those in bulk soil and were dominated by Gram-negative *Proteobacteria* (Fig. 2). This result was observed in all four sampling sites, despite the significantly different soil environmental factors, and suggest that mycorrhizospheres have a strong influence on culturable soil bacterial communities. *Paraburkholderia* was the major genus associated with ECM root tips (Fig. 2B). Previous studies reported that *Burkholderia* is among the predominant genera in the mycorrhizospheres of several species of pine trees, including *P. contorta* (8), *P. muricata* (28), *P. sylvestris* (25), and *P. thunbergii* (20). In recent years, many species of *Burkholderia* have been reclassified as *Paraburkholderia* or *Caballeronia* (12, 34). For example, *B. phenazinium* and *B. sordidicola*, often found in the mycorrhizosphere of *P. muricata* (28), have been transferred to the genus *Paraburkholderia* (34). Hence, many species, confirmed by previous studies to be commonly found in the mycorrhizosphere of *Pinus* trees, are now considered to be *Paraburkholderia*.

According to the NMDS ordination, no significant differences between culturable bacterial communities were evident regardless of the species of ECM fungi (classified into 18 MOTUs). *Paraburkholderia* was found in most of the root tips; however, its proportions in different culturable bacterial communities were variable (Fig. 3). The effects of specific fungal symbionts have been reported in previous studies. Kataoka *et al*. (20) showed that the ECMs, *Russula* spp. and *Suillus* sp., harbored fewer bacteria than *Cenococcum geophilum* in *P. thunbergii*. Marupakula *et al*. (25) also demonstrated the variability of bacterial communities of *P. sylvestris* roots colonized by *Meliniomyces* sp., *Paxillus* sp., and *Russula* sp. These studies were consistent in their finding that certain genera including *Burkholderia* (*Paraburkholderia*) were present in high numbers. These findings suggest that different ECM fungal species have common (rather than species-specific) effects on bacterial communities.

Antibacterial assays indicated that four of the six strains of ECM fungal mycelia exhibited bacteriostatic activity against Gram-positive bacteria. The level of antibacterial activity was variable in the different fungal strains. However, they all displayed a similar antibacterial spectrum (Table 3). Previous studies using sporocarp extracts indicated that ECM fungal species including *Lactarius deliciosus*, *Sarcodon imbricatus*, and *Tricholoma portentosum*, which were not tested in the present study, also showed similarities in their behavior towards bacteria despite differences in their minimum inhibitory concentrations (MICs) (2, 3). These findings suggest that the antibacterial activity of ECM fungi is effective against Gram-positive bacteria and is similar among ECM fungal species. *A. pantherina* and *R. mariae* displayed little or no antibacterial activity. This may be attributed to the low density of mycelia. *A. pantherina* was able to inhibit the growth of *Bacillus* sp. 2 when the number of mycelial discs was tripled (data not shown). *R. mariae* exhibited activity against *B. drentensis* strain S-s330 in the preliminary assay (Table S1). Thus, the strains that did not display antibacterial activity in this test may also exert antibacterial effects.

Co-culture testing demonstrated that Gram-positive bacteria were sensitive to the antibacterial activity of ECM fungi, whereas Gram-negative bacteria were not (Table 3). These differences between Gram-negative and -positive bacterial sensitivities may be attributed to differences in membrane structures. Gram-negative bacteria possess an outer membrane and a periplasmic space, whereas Gram-positive bacteria do not (4). Furthermore, the expression of drug efflux pumps (14) and secretion of an exopolysaccharide matrix (a primary barrier against antimicrobial agents) (23) by Gram-negative bacteria may also account for these differences in antimicrobial sensitivity. In the present study, the majority of Gram-positive strains were localized in bulk soil, whereas Gram-negative strains were predominantly on the ECM root tips (Fig. 2). These results indicate that Gram-positive bacteria are excluded from the mycorrhizosphere due to their sensitivity to the antibacterial activity of ECM fungi. In effect, the culturable mycorrhizosphere bacterial community is mainly composed of bacteria that are not sensitive to the antibacterial activity of ECM fungi. Further research is required to clarify the potency of antibacterial activity and the degree to which it affects the bacterial community relative to other factors, which include the availability of inorganic nutrients (for certain bacteria grouped under *Proteobacteria*).

We anticipated that the bacterial strains identified in the present study may act as mycorrhiza helpers, particularly because members of the same genera have been reported in previous studies as MHBs (9, 16). However, we found that the majority of bacterial strains exerted a negative effect on the growth of the six ECM fungi tested (Table 4). Interestingly, the degree of hyphal growth inhibition was greater in strains that were not sensitive to the antibacterial activity of ECM fungi. For example, most isolates of *Proteobacteria*, which are abundant in the mycorrhizosphere, strongly suppressed hyphal growth. Two strains of *Bacillus* (*Bacillus* sp. 3 and 7), which were not sensitive to all the ECM fungi tested, also significantly inhibited mycelial growth. These results suggest that bacterial sensitivity to the co-cultured ECM fungus and the inhibitory effects of the bacteria on the ECM fungus may be related. The antifungal activity of mycorrhizosphere bacteria is also dependent on the nutritional condition of the medium (32). Root-associated bacteria produce soluble compounds as well as volatile organic compounds (16, 35); however, our experiments are only indicative of effects mediated by soluble compounds. Thus, further studies are required to assess the negative effects of bacteria on ECM fungi.

In conclusion, our investigation on mycorrhizosphere bacteria and the antibacterial activity of ECM fungi has addressed a gap in knowledge in this field. We demonstrated that bacterial strains that are not sensitive to the antibacterial activity of ECM fungi are present in high numbers in the mycorrhizosphere. These results indicate that the antibacterial activity of ECM fungi is an important factor involved in the formation of culturable bacterial communities in the mycorrhizosphere.

## Supplementary Information



## Figures and Tables

**Table 1:** Selected trees, elevation, and soil parameters at four sampling sites.

Sampling site	A	B	C	C (seedlings)	D
Elevation (m)	245.4	246.2	261.0	261.0	266.3
Number of selected trees	3 (A1–A3)	5 (B1–B5)	2 (C1–C2)	5 (Cs1–Cs5)	3 (D1–D3)
Average tree height (m)	20.33±3.01	21.50±3.14	20.60	0.13±0.06	21.58±2.04
Average DBH (cm)	28.93±8.48	34.62±10.98	31.95	—	37.3±4.95
Soil parameters (*n*=5)					
SH (kg cm^−2^)	3.60±1.38 a	1.49±0.47 b	1.38±0.60 b	1.38±0.60 b	0.86±0.11 b
LT (mm)	25.6±8.17 a	37.2±13.22 a	24.8±17.28 a	24.8±17.28 a	29.2±10.31 a
SWC (%)	41.43 a	32.96 b	40.34 a	40.34 a	19.98 c
pH	4.62±0.19 ab	4.34±0.26 b	4.92±0.38 a	4.92±0.38 a	3.55±0.16 c
C/N	15.77±0.73 a	19.27±1.39 bc	18.46±2.09 ab	18.46±2.09 ab	22.15±1.88 c

SH=soil hardness, LT=litter thickness, SWC=soil water content, DBH=diameter at breast height.

mean±standard deviation.

Different letters (a, b, c) indicate significant differences between sampling areas, according to Tukey’s test. *p*<0.05.

**Table 2:** ECM fungal molecular operational taxonomic units isolated from *P. densiflora*.

ECM root tip^a^	ECM fungi	bp	match (%)	Best match acc. no.
A1	*Russula* sp.1	514	98	EU569269
A2	*Sebacina epigaea*	512	98	KF000411
A3a	*Russula heterophylla*	510	97	DQ422006
A3b	*Russula* sp.1	514	98	EU569269

B1	*Lactarius* sp.1	325	99	MH984997
B2	*Lactarius* sp.2	386	97	LC013378
B3	*Amanita* sp.	461	95	KU497540
B4a	Uncultured *Trechisporales*	533	96	JF691338
B4b	*Russula* sp.2	516	98	JN129409
B5	*Thelephoraceae* sp.	833	95	FN669257

C1	*Lactarius hatsudake*	504	100	KR364085
C2a	*Tomentella* sp.	503	100	AB848667
C2b	*Tomentella* sp.	503	100	AB848667
C2c	*Rhizopogon flavidus*	493	98	KP893815

Cs1a	*Tomentella cinerascens*	504	97	AF272915
Cs1b	*Tomentella* sp.	503	100	AB848667
Cs2	*Clitopilus* sp.1	492	97	KU180453
Cs3	unknown^b^	—	—	—
Cs4a	*Rhizopogon* sp.1	307	96	AF062936
Cs4b	*Rhizopogon* sp.1	307	96	AF062936
Cs5	*Rhizopogon* sp.2	483	99	AB253521

D1a	unknown^b^	—	—	—
D1b	unknown^b^	—	—	—
D2	*Sebacina incrustans*	401	98	JQ665543
D3	*Tomentellopsis zygodesmoides*	486	98	AJ410760

a The first letter (A–D) represents the sampling site (a lowercase “s” represents seedling), and the numbers 1–5 represent the serial numbers of mature trees or seedlings in each sampling area.

b Indicates ECM fungi that could not be identified.

**Table 3:** Mean diameter of inhibition zones of 21 bacterial strains due to antibacterial activities of six ECM fungal strains in co-culture testing (*n*=3).

Strains	Zone of inhibition (mm)						Gram	Source of isolation	acc. no.

	*S. bovinus*	*H. mesophaeum*	*R. roseolus*	*A. pantherina*	*S. granulatus*	*R. mariae*			
*Arthrobacter* sp.	9.67±1.01	—	—	—	—	—	p	Bulk soil	LC435746
*Bacillus* sp.1	12.83±0.63	14.50±1.41	—	—	—	—	p	Bulk soil	LC435747
*Bacillus* sp.2	15.58	15.33±0.88	11.33±1.15	—	—	—	p	Bulk soil	LC435748
*Bacillus* sp.3	—	—	—	—	—	—	p	Bulk soil	LC435749
*Bacillus* sp.4	17.58±4.11	15.92±1.01	12.75±3.03	—	—	—	p	Bulk soil	LC435750
*Bacillus* sp.5	13.17±0.76	15.83±4.04	16.17±3.75	—	—	—	p	Bulk soil	LC435751
*Bacillus* sp.6	16.50±1.80	16.83±2.57	11.50±1.80	—	—	—	p	Bulk soil	LC435752
*Bacillus* sp.7	—	—	—	—	—	—	p	Bulk soil	LC435753
*Bacillus* sp.8	14.33±1.26	15.75±2.47	—	—	—	—	p	ECM	LC435754
*Lysinibacillus* sp.	15.25±1.52	—	10.17±0.58	11.25±0.35	—	—	p	Bulk soil	LC435755
*Paenibacillus* sp.	—	—	—	—	—	—	p	Bulk soil	LC435756

*Burkholderia* sp.1	—	—	—	—	—	—	n	Bulk soil	LC435757
*Burkholderia* sp.2	—	—	—	—	—	—	n	ECM	LC435758
*Collimonas* sp.	—	—	—	—	—	—	n	ECM	LC435759
*Massilia* sp.	—	—	—	—	—	—	n	ECM	LC435760
*Paraburkholderia* sp.1	—	—	—	—	—	—	n	Bulk soil	LC435761
*Paraburkholderia* sp.2	—	—	—	—	—	—	n	Bulk soil	LC435762
*Paraburkholderia* sp.3	—	—	—	—	—	—	n	ECM	LC435763
*Paraburkholderia* sp.4	—	—	—	—	—	—	n	ECM	LC435764
*Paraburkholderia* sp.5	—	—	—	—	—	—	n	ECM	LC435765
*Rhizobium* sp.	—	—	—	—	—	—	n	ECM	LC435766

mean±standard deviation, — no inhibition zone formation.

The characters “p” and “n” denote Gram-positive and -negative bacteria, respectively.

**Table 4:** Mycelial growth of six ECM fungal strains co-cultivated with 21 bacterial strains for four weeks (*n*=3).

Strains	Mycelial growth (mm)						Gram	Source of isolation	acc. no.

	*S. bovinus*	*H. mesophaeum*	*R. roseolus*	*A. pantherina*	*S. granulatus*	*R. mariae*			
control	31.17±3.51	22.83±3.55	57.00±4.09	26.00±3.50	51.67±4.07	13.58±0.14			

*Arthrobacter* sp.	11.83±1.04 **	0	42.33±1.15 **	19.50±1.80 *	26.83±0.76 **	6.50±0.50 **	p	Bulk soil	LC435746
*Bacillus* sp.1	25.83±2.93 N.S.	11.17±0.29 **	56.67±0.76 N.S.	0	42.00±2.78 *	6.67±1.15 **	p	Bulk soil	LC435747
*Bacillus* sp.2	30.00±1.80 N.S.	13.42±0.63 *	58.33±1.53 N.S.	29.33±1.89 N.S.	50.67±2.08 N.S.	10.00±1.73 N.S.	p	Bulk soil	LC435748
*Bacillus* sp.3	1.00	5.17±5.84 *	15.00±8.41 **	0	10.25±0.25 **	0	p	Bulk soil	LC435749
*Bacillus* sp.4	28.33±2.75 N.S.	14.83±3.21 *	55.17±2.02 N.S.	26.67±3.69 N.S.	49.00±4.00 N.S.	6.58±0.63 **	p	Bulk soil	LC435750
*Bacillus* sp.5	28.33±1.26 N.S.	21.67±4.51 N.S.	56.83±0.76 N.S.	26.33±2.57 N.S.	45.67±4.25 N.S.	7.50±0.50 **	p	Bulk soil	LC435751
*Bacillus* sp.6	27.00±1.73 N.S.	15.00±1.32 *	50.50±1.73 N.S.	26.83±4.80 N.S.	42.17±4.01 *	7.92±0.14 **	p	Bulk soil	LC435752
*Bacillus* sp.7	0	0	21.50±0.50 **	0	5.67±5.30 **	0	p	Bulk soil	LC435753
*Bacillus* sp.8	31.67±3.33 N.S.	19.67±0.58 N.S.	10.83±1.15 **	0	47.08±4.64 N.S.	9.42±0.52 **	p	ECM	LC435754
*Lysinibacillus* sp.	28.83±0.76 N.S.	27.67±3.51 N.S.	32.67±5.77 **	0	43.50±1.50 *	10.33±0.29 **	p	Bulk soil	LC435755
*Paenibacillus* sp.	1.00	0.67±0.58 **	49.00±3.61 N.S.	33.33±0.29 *	5.33±0.29 **	0	p	Bulk soil	LC435756

*Burkholderia* sp.1	0	0	13.50±0.87 **	0	5.17±5.84 **	0	n	Bulk soil	LC435757
*Burkholderia* sp.2	5.17±1.04 **	0	26.50±5.22 **	3.25±1.06 **	27.92±2.32 **	0	n	ECM	LC435758
*Collimonas* sp.	10.83±1.53 **	2.50±0.43 **	14.67±4.86 **	0	54.08±2.24 N.S.	4.42±0.72 **	n	ECM	LC435759
*Massilia* sp.	0	0	14.50±0.50 **	0.75±0.35 **	17.58±0.29 **	0	n	ECM	LC435760
*Paraburkholderia* sp.1	3.17±2.84 **	0	23.50±4.82 **	0	9.92±11.48 **	0	n	Bulk soil	LC435761
*Paraburkholderia* sp.2	0	4.67±4.51 **	5.00±2.29 **	0	12.17±3.40 **	0	n	Bulk soil	LC435762
*Paraburkholderia* sp.3	6.33±2.02 **	0	29.33±2.36 **	2.00±1.32 **	17.83±3.06 **	0	n	ECM	LC435763
*Paraburkholderia* sp.4	0	4.33±0.76 **	28.83±5.39 **	0	9.33±2.08 **	0	n	ECM	LC435764
*Paraburkholderia* sp.5	16.67±1.44 **	0	32.33±2.93 **	12.83±0.29 **	39.00±3.12 *	0	n	ECM	LC435765
*Rhizobium* sp.	0	0	30.50±5.22 **	0	0.70±0.58 **	0	n	ECM	LC435766

mean±standard deviation.

The characters “p” and “n” denote Gram-positive and -negative bacteria, respectively.

Significant differences in mycelial growth between mono-cultivated ECM fungi and those co-cultivated with bacteria were examined using the Student’s *t*-test. N.S., not significant,

* *P*<0.05

** *P*<0.01.
