# Supplementary material for: Mycorrhizosphere Bacterial Communities and their Sensitivity to Antibacterial Activity of Ectomycorrhizal Fungi
Source: Microbes Environ. 2019 May 11;34(2):191–8. doi: 10.1264/jsme2.ME18146 (PMC6594744; doi:10.1264/jsme2.ME18146)
Supplement: Supplementary file 1 [file 34_191_s1.pdf]

**Table S1.** Antibacterial activity of 33 ECM fungal strains against *B. drentensis* strain S-s330 (*n*=3).

| Strains | Zone of inhibition (mm) |
|---|---|
| *Amanita pantherina* | - |
| *Amanita rubescens* | - |
| *Amanita virgineoides* | - |
| *Boletinus cavipes* | 15.50±0.66 |
| *Cenococcum geophilum* 1 | 20.92±3.02 |
| *Cenococcum geophilum* 2 | 9.83±2.57 |
| *Cortinarius purpurascens* | - |
| *Hebeloma leucosarx* | 13.25±1.50 |
| *Hebeloma mesophaeum* | 20.25±4.21 |
| *Laccaria amethystea* | 16.33±3.33 |
| *Laccaria laccata* 1 | 9.75±1.39 |
| *Laccaria laccata* 2 | 8.83±0.95 |
| *Laccaria murina* | 13.00±0.50 |
| *Pisolithus* sp. 1 | 13.75±3.50 |
| *Pisolithus* sp. 2 | 19.50±0.66 |
| *Pisolithus* sp. 3 | 20.08±1.66 |
| *Rhizopogon roseolus* | 11.67±0.76 |
| *Russula mariae* | 9.00±1.00 |
| *Russula pectinatoides* | - |
| *Russula sanguinaria* | - |
| *Russula sororia* | - |
| *Russula violeipes* | - |
| *Scleroderma bovista* | - |
| *Suillus bovinus* | 17.67±0.29 |
| *Suillus granulatus* | - |
| *Suillus grevillei* 1 | 20.42±1.94 |
| *Suillus grevillei* 2 | 10.08±1.13 |
| *Suillus laricinus* | 12.33±1.66 |
| *Suillus luteus* | - |
| *Suillus spectabilis* | 16.50±0.50 |
| *Tricholoma matsutake* 1 | - |
| *Tricholoma matsutake* 2 | - |
| *Tricholoma psammopus* | 18.83±1.76 |

- no inhibition zone formation.
Gray color indicates the strains used for antibacterial assays.

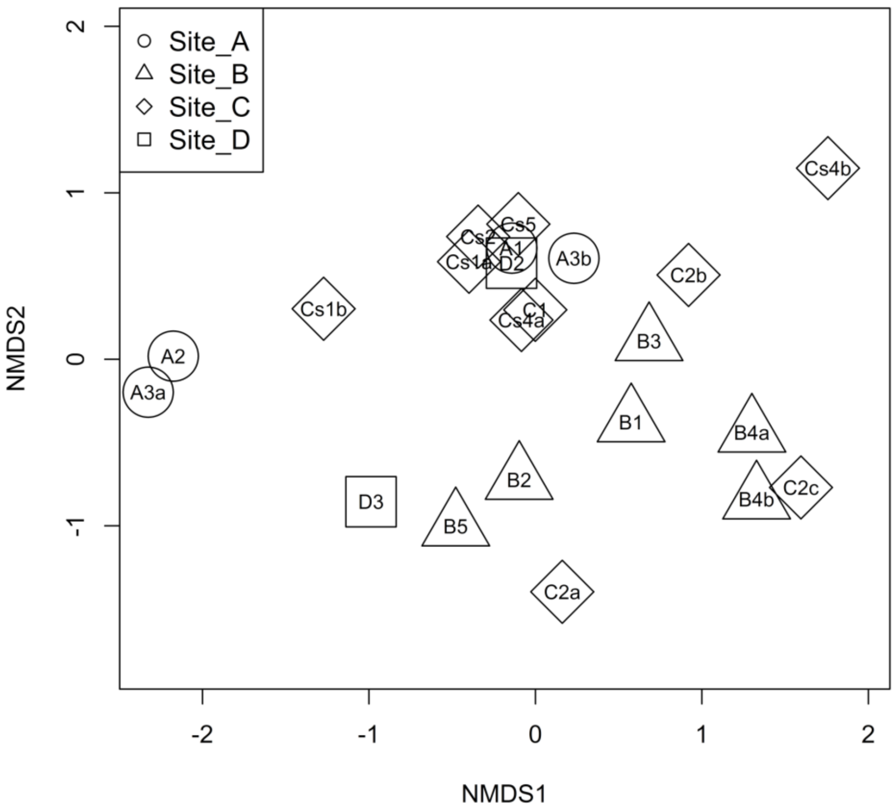


**Fig. S1.** Non-metric multidimensional scaling (NMDS) ordination of the culturable bacterial community composition associated with ECM root tips of *P. densiflora*. Circles, triangles, diamonds, and squares indicate four sampling sites A–D, respectively. The first letter (A–D) represents the sampling sites (lowercase "s" represents a seedling), and the numbers 1–5 represent serial numbers of mature trees or seedlings in each sampling area. Stress=0.1113.
